# Visual impairment and multimorbidity in a representative sample of the Spanish population

**DOI:** 10.1186/1471-2458-14-815

**Published:** 2014-08-08

**Authors:** Noe Garin, Beatriz Olaya, Elvira Lara, Maria Victoria Moneta, Marta Miret, Jose Luis Ayuso-Mateos, Josep Maria Haro

**Affiliations:** Parc Sanitari Sant Joan de Déu, Universitat de Barcelona, Dr Antoni Pujades, 42, 08830 Barcelona, Sant Boi de Llobregat, Spain; Fundació Sant Joan de Déu, Santa Rosa, 39-57, 08950 Barcelona, Esplugues de Llobregat, Spain; Instituto de Salud Carlos III, Centro de Investigación Biomédica en Red de Salud Mental, CIBERSAM, Monforte de Lemos 3–5. Pabellón 11, 28029 Madrid, Spain; Department of Psychiatry, Universidad Autónoma de Madrid, Arzobispo Morcillo s/n, 28029 Madrid, Spain; Instituto de Investigación Sanitaria Princesa (IP), Diego de León, 62, 28006 Madrid, Spain

**Keywords:** Epidemiology, Chronic conditions, Multimorbidity, Visual impairment, Low vision, Mental health, Cognitive functioning, Elderly

## Abstract

**Background:**

In the context of population aging, visual impairment has emerged as a growing concern in public health. However, there is a need for further research into the relationship between visual impairment and chronic medical conditions in the elderly. The aim of our study was to examine the relationship between visual impairment and three main types of co-morbidity: chronic physical conditions (both at an independent and additive level), mental health and cognitive functioning.

**Methods:**

Data were collected from the COURAGE in Europe project, a cross-sectional study. A total of 4,583 participants from Spain were included. Diagnosis of chronic medical conditions included self-reported medical diagnosis and symptomatic algorithms. Depression and anxiety were assessed using CIDI algorithms. Visual assessment included objective distance/near visual acuity and subjective visual performance. Descriptive analyses included the whole sample (n = 4,583). Statistical analyses included participants aged over 50 years (n = 3,625; mean age = 66.45 years) since they have a significant prevalence of chronic conditions and visual impairment. Crude and adjusted binary logistic regressions were performed to identify independent associations between visual impairment and chronic medical conditions, physical multimorbidity and mental conditions. Covariates included age, gender, marital status, education level, employment status and urbanicity.

**Results:**

The number of chronic physical conditions was found to be associated with poorer results in both distance and near visual acuity [OR 1.75 (CI 1.38-2.23); OR 1.69 (CI 1.27-2.24)]. At an independent level, arthritis, stroke and diabetes were associated with poorer distance visual acuity results after adjusting for covariates [OR 1.79 (CI 1.46-2.21); OR 1.59 (CI 1.05-2.42); OR 1.27 (1.01-1.60)]. Only stroke was associated with near visual impairment [OR 3.01 (CI 1.86-4.87)]. With regard to mental health, poor subjective visual acuity was associated with depression [OR 1.61 (CI 1.14-2.27); OR 1.48 (CI 1.03-2.13)]. Both objective and subjective poor distance and near visual acuity were associated with worse cognitive functioning.

**Conclusions:**

Arthritis, stroke and the co-occurrence of various chronic physical diseases are associated with higher prevalence of visual impairment. Visual impairment is associated with higher prevalence of depression and poorer cognitive function results. There is a need to implement patient-centered care involving special visual assessment in these cases.

## Background

Visual impairment is related to higher morbidity, increased risk of falling, activity limitations, lower quality of life, poor social participation and increased mortality [1–8]. Low vision is a state of moderate or severe visual impairment defined as a visual acuity lower than 6/18 and equal to or better than 3/60 in the better eye with best correction [9]. Even though this represents significant impairment, people affected with low vision are still potentially able to perform some activities for which sight is essential. The relevance of vision to health status has led the World Health Organization to recently introduce vision and other sensory functions as health domains in the International Classification of Functioning, Disability and Health (ICF), a unified framework for a description of health that highlights the interaction between health conditions and contextual factors [10].

In industrialized countries, the main causes of low vision and blindness are age-related macular degeneration (AMD), glaucoma, cataracts, pathologic myopia and diabetic retinopathy. The prevalence of all these disorders increases with age [11, 12]. In fact, about 65% of people who are visually impaired are aged 50 and older, while this group only represents about 20% of the world’s population [13]. Since a 2-fold increase in the population over 60 years old is expected between 2006 and 2050, the number of people with impaired vision is expected to increase in the future [14, 15]. Although some of these disorders can be treated, for instance through cataract surgery or AMD intravitreal pharmacotherapy, permanent functional visual impairment can appear in cases of delayed or inefficacious treatment [16].

Aging is also clearly associated with the onset of chronic physical and mental conditions and an increase in multimorbidity [17, 18]. According to a recent review, between 55% and 98% of the elderly population suffer from multimorbidity [19]. Some particular associations have been identified between chronic physical conditions and vision disorders, such as diabetes and retinopathy [20]. However, there is very limited information on the relationship between visual functioning and most common medical chronic conditions at both the independent and additive levels. Sensory impairment has also been linked to psychological and social problems. Mild or moderate visual impairment in adults have been found to be associated with mental distress, depression, anxiety, suicide risk, interpersonal sensitivity and hostility [2, 21–26]. Most of these studies have focused on depression and little is known about the relationship between sensorial impairment and other psychiatric conditions, such as anxiety, especially in the elderly. Moreover, there is evidence of an association between visual impairment and dementia [27, 28]. To date, however, research focusing specifically on the association between visual impairment and cognitive functioning has showed mixed results [29–33]. Determining the relationship between visual impairment and chronic conditions through a comprehensive approach might contribute to the development of optimal patient-centered care to the elderly.

We used a large general population survey to examine:

- The relationship between physical multimorbidity and visual impairment in the population over 50 years old.
- The relationship between visual impairment and mental health (depression and anxiety) in the population over 50 years old.
- The relationship between visual impairment and cognitive functioning in the population over 50 years old.

## Methods

### Design

The COURAGE in Europe project is a cross-sectional household survey of a representative sample of the non-institutionalized adult population conducted in Finland, Poland and Spain [34]. Data from the Spanish sample are analyzed in the current study.

### Sample and procedures

A representative sample of the adult population in Spain was selected using a stratified multistage clustered area probability method. The target group was a community-residing population over 18 years. Three samples were selected according to age: 18–49; 50–79; ≥80 years. The 50+ and 80+ subgroups were oversampled as they were the main target of the study. People with language barriers were excluded. From July 2011 to May 2012, face-to-face structured interviews were conducted through Computer-Assisted Personal Interviewing (CAPI) at respondents’ homes. The survey protocol was translated from English into Spanish according to WHO translation guidelines for assessment instruments [35]. Lay interviewers were trained before administration of the survey. Quality assurance procedures were implemented during fieldwork [36]. The final response rate was 69.9%. The main reason for non-response was that the house was unoccupied or that the members of the household were elsewhere (seasonal vacancy, other residence). The interviewer judged whether the respondent had cognitive problems at the beginning of the interview. If cognitive difficulties were evident, a short version of the survey was obtained from proxy respondents. Data from proxy respondents were not analyzed since visual assessment and diagnosis of chronic physical conditions and mental disorders were not performed in the proxy interviews. Therefore, once the 170 proxy respondents had been eliminated, the final analysis consisted of 4,583 participants. This sample was used for initial descriptive purposes. The final analysis consisted of 3,625 participants over 50 years old since this subgroup suffers from significant prevalence of chronic conditions and visual impairment compared with younger populations.

### Data collection

Sociodemographic information included age, gender, marital status, education level, employment status and urbanicity. With regard to chronic physical conditions, participants were asked whether they had received life-time medical diagnosis and treatment during the previous 12 months for angina, arthritis, asthma, chronic lung disease, diabetes, edentulism, hypertension and stroke. Moreover, algorithms based on clinical symptoms were implemented to detect undiagnosed cases. These were based on the WHO’s SAGE study, current clinical guidelines and reference publications [37–43]. In the case of meeting at least one of the two previously established criteria, the respondent was considered to have angina, asthma, arthritis, chronic lung disease or stroke. Hypertension, diabetes and edentulism are asymptomatic conditions so no symptomatic algorithm was used. Previous 12-month major depressive disorder and anxiety disorders (including Generalized Anxiety Disorder and Panic Disorder) were assessed with an adapted version of the World Health Organization Composite International Diagnostic Interview (CIDI), according to DSM-IV criteria [44]. Cognitive functioning results were calculated from five performance tests in distinct domains: learning and short-term memory (word list immediate and delayed recall from the Consortium to Establish a Registry for Alzheimer’s Disease), attention and working memory (digit spans forward and backward from the Weschler Adult Intelligence Scale) and language (animal naming task) [45, 46]. All performance tests were scored according to standard practice for each test. A global score was calculated using the sum of the standardized score on each item, with a lower score indicating worse cognitive functioning [47]. Analogous methodology for computing this score with the same tests has previously been used in the WHO’s SAGE and COURAGE studies [47, 48]. In our case, we also standardized results by level of education. Results were later transformed into a binary variable for analysis purposes. Objective visual acuity was measured by the interviewer with a “tumbling E” chart [49]. To assess distance vision, an appropriate chart was placed 3 meters from the participant. Visual acuity was registered using the metric visual acuity scale, through which patients were classified into one of the following groups: visual acuity less than 3/60, 3/60-6/60, 6/60-6/18 and more than 6/18. To demonstrate the visual acuity required by a line in the chart, the participant had to detect at least three of the four letters in the line. Distance VA was measured initially for the left eye and subsequently for the right eye. Analog procedures were conducted to assess near vision, with a specific appropriate chart that was held by the participant at a comfortable distance. For near vision, participants were classified as: unable to see the largest sized letter (<N48), able to see largest size (N48), able to see medium size (N20), able to see the smallest size (N8). All the measures were taken in daily-life light conditions with the participant’s usual visual correction. The interviewer made sure that the vision chart was well lit with natural or indoor lighting and that the surface did not reflect glare. Visual performance for distance and near vision was taken from the eye with the better visual acuity results. Subjective visual acuity was assessed through two questions: a) *In the last 30 days, how much difficulty did you have in seeing and recognizing an object or a person you know across the road (from a distance of about 20 meters)?*; b) *In the last 30 days, how much difficulty did you have in seeing and recognizing an object at arm’s length (for example, reading)?* Five answers were permitted: none, mild, moderate, severe, extreme.

### Statistical analysis

Unweighted frequencies, weighted proportions, means, confidence intervals and cross tabulations were applied for descriptive analyses. The Chi-square test was used to measure differences in visual performance, prevalence of chronic diseases, multimorbidity, number of conditions and sociodemographic variables across age and gender.

Crude and adjusted binary logistic regressions were used to examine the association between chronic physical conditions and visual performance in participants over 50 years (n = 3625). Results are reported as Odds Ratio (OR) with 95% CI. Adjusted models included age, gender, education level, marital status, urbanicity, and all chronic physical conditions. For the analyses regarding the relationship between multimorbidity and visual performance, adjusted models included age, gender, education level, marital status, urbanicity, and number of chronic conditions. Analogous procedures were used to examine the association between visual acuity and the variables related to mental health (depression, anxiety) and cognitive functioning. In this case, adjusted models included age, gender, education level, marital status, urbanicity and all chronic physical conditions. Visual acuity variables were transformed into binary variables for the logistic regression. Objective distance visual acuity was classified as “poor” (lower than 6/18) or “good” (equal or better than 6/18). Objective near visual acuity was classified as “good” (able to read the smallest letters) or “poor” (other cases). Subjective visual acuity with no or mild problems was considered “good”, and “poor” in the case of more serious problems. The Kappa inter-agreement test was performed for objective and subjective visual performance. The association was considered as fair (Kappa: 0.3206; Standard Error: 0.0118) [50]. For this reason, objective and subjective visual performance were taken into account separately in the logistic regression relating vision and mental health.

The statistical analyses took into consideration the complex nature of the sample design. Weights were used in all analyses to adjust for differential probabilities of selection within households, and post-stratification weights to match the samples to population socio-demographic distributions. Analyses were performed using IBM SPSS statistics 19.

### Ethics statement

The COURAGE study was approved by the partners’ Ethics Committees: Fundació Sant Joan de Déu Ethics Review Committee, Barcelona, Spain and La Princesa University Hospital Ethics Review Committee, Madrid, Spain. Written informed consent was obtained from the participants. All investigators worked according to the principles expressed in the Declaration of Helsinki.

## Results

### Participant characteristics

The study population consisted of 4,583 participants. A summary of the sociodemographic data is available in Table 1. Age differences are statistically significant in educational level, marital status and employment. As expected, prevalence of physical multimorbidity, chronic physical conditions and mental conditions increased with age, except for anxiety (Table 2). Cognitive functioning below median per group was: a) 18–49 years: 48.5% (CI: 44.9-52.1); b) 50–64 years: 65.2% (CI: 61.5-68.7); c) 65+: 83.1% (CI: 80.9-85.0).

**Table 1:** **Description of the sample of the Spanish Cohort of the COURAGE study**

	Total sample (n = 4583)	18-49 years (n = 958)	50-64 years (n = 1760)	≥65 years (n = 1865)	*p*
Age (mean; SE)	47.6 (0.3)	35.7 (0.30)	57.0 (0.1)	74.9 (0.1)	
Education (n; %)					<0.001
No education	1269 (16.64%)	62 (6.53%)	311 (16.62%)	896 (46.70%)	
Primary	1265 (24.93%)	190 (20.92%)	548 (32.81%)	527 (29.84%)	
Secondary	1423 (39.29%)	474 (48.07%)	638 (35.42%)	311 (16.61%)	
≥University	625 (19.14%)	232 (24.48%)	263 (15.15%)	130 (6.80%)	
Gender (n; %)					0.006
Male	2078 (49.4%)	435 (51.35%)	829 (47.70%)	814 (44.95%)	
Female	2505 (50.6%)	523 (48.65%)	931 (52.30)	1051 (55.04%)	
Marital status (n; %)					<0.001
Single	667 (27.3%)	357 (39.18%)	187 (10.39%)	123 (6.75%)	
Married	2777 (56.6%)	519 (53.12%)	1238 (71.10%)	1020 (54.18%)	
Separated/divorced	342 (7.1%)	76 (7.21%)	196 (10.86%)	70 (3.36%)	
Widowed	797 (9.0%)	6 (0.49%)	139 (7.64%)	652 (35.72%)	
Urbanicity (n; %)					0.216
Urban	3958 (84.78%)	820 (85.40%)	1523 (83.69%)	1615 (83.93%)	
Rural	625 (15.22%)	138 (14.60%)	237 (16.31%)	250 (16.07%)	
Employment (n; %)					<0.001
Working	1360 (46.21%)	543 (61.34%)	773 (47.47%)	44 (2.28%)	
Retired	1387 (16.43%)	2 (0.24%)	183 (11.35%)	1202 (66.56%)	
Other	1611 (37.36%)	342 (38.41%)	681 (41.18%)	588 (31.16%)	

SE = Standard Error.

Note = unweighted frequencies (n), and weighted means and proportions are displayed. Household income was divided into 5 quintiles. Education category ‘no education’ included those people that had never been to school or did not finish primary school. Marital status ‘married’ category included currently married or cohabiting. Employment ‘other’ category included training, homemakers, unemployed, voluntary work, health problems, caring for family, sick leave, no need to work, temporary time off and voluntary work.

**Table 2:** **Prevalence of 12-month chronic physical conditions, mental disorders and multimorbidity according to age**

	Total	18-49 years	50-64 years	≥65 years	*p*(age)
**Angina**	2.84 (2.44-3.29)	0.4 (0.2-0.9)	3.8 (2.9-4.9)	9.2 (7.9-10.7)	<0.001
**Asthma**	4.83 (4.11-5.67)	3.9 (2.9-5.1)	4.3 (3.5-5.4)	8.1 (6.7-9.7)	<0.001
**Hypertension**	16.55 (15.48-17.68)	3.4 (2.3-5.0)	23.4 (21.3-25.7)	49.7 (47.4-52.0)	<0.001
**Edentulism**	9.03 (7.67-10.60)	2.8 (1.6-5.0)	8.2 (6.8-9.9)	28.3 (25.0-31.8)	<0.001
**Diabetes**	6.66 (5.95-7.45)	2.0 (1.3-3.0)	9.5 (7.9-11.3)	18.1 (16.2-20.1)	<0.001
**Arthritis**	13.72 (12.66-14.84)	5.4 (4.2-7.0)	18.4 (16.4-20.6)	34.3 (31.8-36.9)	<0.001
**Chronic Lung D**	3.35 (2.83-3.95)	1.2 (0.7-2.1)	4.0 (3.1-5.1)	9.1 (7.7-10.8)	<0.001
**Stroke**	2.13 (1.71-2.64)	0.5 (0.3-1.2)	2.4 (1.6-3.5)	6.6 (5.3-8.3)	<0.001
**Depression**	8.97 (7.88-10.19)	7.0 (5.5-8.8)	12.0 (9.8-14.5)	12.2 (10.6-13.9)	<0.001
**Anxiety**	1.09 (0.77-1.54)	0.9 (0.5-1.7)	1.8 (1.3-2.5)	1.0 (0.7-1.6)	0.120
**Number of physical conditions**					
**0**	64.4 (62.7-66.1)	82.8 (80.3-85.0)	53.0 (49.4-56.5)	19.9 (17.9-22.1)	<0.001
**1**	21.3 (19.9-22.7)	15.3 (13.2-17.7)	29.7 (27.3-32.3)	31.5 (29.7-33.4)	<0.001
**2**	8.1 (7.5-8.8)	1.4 (0.9-2.2)	10.3 (8.8-12.1)	26.3 (24.2-28.5)	<0.001
**≥3**	6.2 (5.6-6.8)	0.5 (0.2-1.2)	7.0 (5.8-8.3)	22.3 (20.1-24.7)	<0.001

Note = Weighted proportion and 95% Confidence Intervals are shown. Anxiety included Generalized Anxiety Disorder and Panic Disorder.

In the overall adult population, a decrease in distance visual acuity was observed across age groups (Table 3). No gender differences were observed. For near visual acuity, the worst results were found in the elderly and in women. In the population aged over 65 years, only 33.8% of the women and 43.0% of the men achieved highest near visual acuity. Age and gender differences were also present in the subjective perception of distance and near vision problems. Elderly women was the group with the poorest results.

**Table 3:** **Vision acuity results across age and sex groups**

	TOTAL	18-49 years		50-64 years		> 65 years		*p*(age)	*p*(sex)
		Men	Women	Men	Women	Men	Women		
**Objective DVA**								<0.001	0.113
≥6/18	79.7 (76.3-82.8)	81.6 (76.0-86.1)	86.2 (82.2-89.4)	83.2 (79.8-86.1)	79.7 (75.2-83.7)	69.4 (64.3-74.0)	62.5 (57.7-67.1)		
6/60	16.7 (13.9-20.0)	17.0 (12.6-22.4)	11.9 (8.9-15.6)	15.1 (12.3-18.3)	17.7 (14.2-21.9)	21.9 (18.3-26.1)	25.3 (21.6-29.5)		
3/60	2.9 (2.3-3.8)	1.4 (0.5-3.9)	1.6 (0.8-3.2)	1.7 (1.0-2.9)	2.3 (1.5-3.7)	7.0 (5.1-9.4)	9.3 (7.4-11.7)		
<3/60	0.6 (0.4-0.9)	0.1 (0.0-0.7)	0.3 (0.1-1.6)	0.1 (0.0-0.4)	0.2 (0.1-0.5)	1.7 (1.0-3.0)	2.8 (1.8-4.5)		
**Objective NVA**								<0.001	<0.001
Smallest letter	68.8 (66.4-71.1)	84.7 (81.0-87.99	80.0 (76.5-83.1)	59.6 (55.8-63.3)	53.7 (49.1-58.3)	43.0 (38.0-48.1)	33.8 (30.1-37.6)		
Medium letter	28.6 (26.5-30.9)	14.5 (11.5-18.2)	18.6 (15.6-22.0)	38.1 (34.4-41.9)	43.4 (39.2-47.8)	50.8 (46.0-55.5)	57.9 (53.6-62.1)		
Large letter	2.1 (1.6-2.7)	0.5 (0.2-1.7)	1.4 (0.6-2.9)	2.1 (1.3-3.3)	2.3 (1.4-3.8)	5.1 (3.9-6.8)	5.9 (4.5-7.7)		
Not large letter	0.5 (0.3-0.8)	0.2 (0.0-1.4)	0	0.2 (0.1-0.8)	0.5 (0.2-1.4)	1.1 (0.5-2.4)	2.5 (1.5-4.1)		
**Subjective DVA**								<0.001	<0.001
None	87.8 (86.3-89.3)	93.2 (89.7-95.6)	93.4 (90.9-95.3)	89.9 (87.2-92.0)	83.9 (80.4-86.8)	78.5 (74.8-81.8)	67.5 (62.8-71.9)		
Mild	8.3 (7.1-9.6)	5.4 (3.4-8.5)	5.0 (3.4-7.2)	8.2 (6.2-10.9)	12.0 (9.5-15.1)	14.1 (11.6-16.9)	17.0 (13.9-20.6)		
Moderate	2.8 (2.3-3.5)	0.8 (0.2-3.4)	1.4 (0.8-2.6)	1.5 (0.9-2.5)	3.2 (2.2-4.5)	5.7 (4.2-7.6)	10.7 (8.8-13.1)		
Severe	1.1 (0.8-1.4)	0.6 (0.2-1.5)	0.2 (0.2-0.2)	0.4 (0.2-1.1)	1.0 (0.5-1.9)	1.8 (1.1-2.9)	4.7 (3.5-6.3)		
**Subjective NVA**								<0.001	0.506
None	89.2 (87.7-90.6)	93.1 (89.7-95.4)	96.2 (94.5-97.3)	88.3 (85.5-90.6)	85.7(82.0-88.8)	81.4 (77.7-84.7)	70.5 (65.6-74.9)		
Mild	8.0 (6.8-9.4)	5.4 (3.4-8.6)	3.2 (2.1-4.8)	8.6 (6.6-11.2)	11.4 (8.7-15.0)	13.3 (10.8-16.3)	19.9 (16.4-24.1)		
Moderate	2.2 (1.7-2.9)	1.0 (0.3-3.5)	0.6 (0.3-1.6)	2.8 (1.7-4.6)	2.6 (1.7-3.8)	4.4 (3.1-6.3)	7.3 (5.6-9.4)		
Severe	0.5 (0.4-0.8)	0.5 (0.2-1.4)	0	0.3 (0.1-0.9)	0.3 (0.1-0.8)	0.8 (0.4-1.7)	2.3 (1.5-3.5)		

DVA = distance visual acuity; NVA: near visual acuity. NOTE = Subjective visual assessment results were related to problems: none, mild problems, moderate problems, severe problems. Weighted proportion and 95% Confident intervals are shown; *p*(age) refers to statistical differences in the three age groups; *p*(sex) refers to differences in gender, with of the age group; subjective visual acuity is assessed as vision problems mentioned by the patient.

### Association between physical health and visual acuity

Table 4 shows the crude and adjusted logistic regression odds ratios for the association between visual acuity and physical medical conditions in individuals over 50 years old (n = 3,625). In the crude analysis, all chronic physical conditions except chronic lung disease were associated with worse distance visual acuity. In the adjusted model, arthritis, stroke and diabetes were still associated with worse distance visual acuity after adjusting for other covariates (OR 1.79 [CI 1.46-2.21]; OR 1.59 [CI 1.05-2.42]; OR 1.27 [1.01-1.60] respectively). Similar results were found in the analysis of near visual acuity, although only stroke resulted in a higher odds of worse visual acuity (OR 3.01 [CI 1.86-4.87]) after adjusting for covariates. The number of concurrent chronic physical conditions was associated with an increased odds of worse visual acuity for both near and distance vision. In the adjusted model for distance visual acuity, patients with three or more chronic physical conditions had the highest odds (OR 1.75 [CI 1.38-2.23]). Similar results were found with regard to the number of chronic physical conditions and near visual acuity. The highest OR was associated with having three or more chronic diseases (OR 1.69 [CI 1.27-2.24]).

**Table 4:** **Association between chronic physical conditions and poor visual acuity**

	DVA (OR)	DVA (AOR)	NVA (OR)	NVA (AOR)
**Angina**	1.57 (1.17-2.11)	1.12 (0.84-1.51)	1.43 (1.07-1.91)	1.07 (0.78-1.48)
**Asthma**	1.42 (1.04-1.95)	1.02 (0.72-1.44)	1.38 (0.99-1.93)	1.04 (0.70-1.54)
**Hypertension**	1.28 (1.08-1.52)	0.87 (0.73-1.04)	1.51 (1.23-1.78)	1.10 (0.94-1.28)
**Edentulism**	1.70 (1.33-2.18)	1.12 (0.89-1.43)	1.74 (1.42-2.14)	1.16 (0.96-1.41)
**Diabetes**	1.59 (1.28-1.98)	1.27 (1.01-1.60)	1.22 (0.86-1.75)	1.15 (0.86-1.52)
**Arthritis**	2.30 (1.92-2.75)	1.79 (1.46-2.21)	1.63 (1.33-1.98)	1.16 (0.97 -1.40)
**Chronic Lung D**	1.38 (0.99-1.93)	1.02 (0.74-1.42)	1.34 (0.90-2.02)	1.06 (0.70-1.60)
**Stroke**	1.87 (1.24-2.83)	1.59 (1.05-2.42)	3.53 (2.18-5.73)	3.01 (1.86-4.87)
**Number of physical conditions**				
**1**	1.67 (1.35-2.08)	1.32 (1.07-1.63)	1.63 (1.38-1.94)	1.33 (1.13-1.57)
**2**	1.95 (1.51-2.51)	1.30 (1.00-1.67)	2.06 (1.64-2.60)	1.43 (1.13-1.81)
**≥3**	2.83 (2.23-3.58)	1.75 (1.38-2.23)	2.64 (2.00-3.48)	1.69 (1.27-2.24)

DVA = distance visual acuity; NVA: near visual acuity; OR = Odds Ratio; AOR = Adjusted Odds Ratio.

Note = Results with 95% Confidence interval. In bold, statistically significant (*p* < 0.05). AORs are based on logistic regression model including all medical conditions, age, gender, educational level, marital status, and urbanicity. For number of physical conditions, the adjusted models included number of physical conditions, age, gender, educational level, marital status, and urbanicity.

### Association between visual acuity and variables related to mental health and cognition

After adjusting for covariates, subjective distance visual acuity and subjective near visual acuity were revealed to be associated with depression (OR 1.61 [CI 1.14-2.27]; OR 1.48 [CI 1.03-2.13]) (Table 5). No association was found in any visual acuity variable with regard to anxiety in the logistic model adjusting for covariates. For cognition, objective distance visual acuity, objective near visual acuity, subjective distance visual acuity and subjective near visual acuity were associated with lower cognitive performance in the adjusted model (OR 1.27 [CI 1.02-1.59]; OR 1.51 [CI: 1.28-1.85]; OR 1.43 [CI 1.00-2.06]; OR 2.40 [CI 1.52-3.71])

**Table 5:** **Association between poor visual acuity and variables related to mental health and cognitive functioning**

	Depression (OR)	Depression (AOR)	Anxiety (OR)	Anxiety (AOR)	Cognition (OR)	Cognition (AOR)
**Distance VA**	1.64 (1.27-2.12)	1.25 (0.97-1.61)	0.90 (0.47-1.71)	0.74 (0.39-1.40)	1.47 (1.17-1.86)	1.27 (1.02-1.59)
**Near VA**	1.39 (1.01-1.93)	1.10 (0.78-1.53)	0.97 (0.55-1.73)	0.83 (0.43-1.64)	1.72 (1.46-2.02)	1.51 (1.28-1.85)
**Subjective distance VA**	2.80 (2.10-3.74)	1.61 (1.14-2.27)	2.15 (1.14-4.04)	1.47 (0.71-3.04)	2.05 (1.44-2.90)	1.43 (1.00-2.06)
**Subjective near VA**	2.40 (1.74-3.30)	1.48 (1.03-2.13)	0.42 (0.09-1.93)	0.27 (0.05-1.36)	2.96 (1.92-4.55)	2.40 (1.52-3.71)

VA = visual acuity; OR = Odds Ratio; AOR = Adjusted Odds Ratio.

Note = Adjusted models included age, gender, education level, marital status, urbanicity and all chronic physical conditions. Results with 95% Confidence interval. In bold, statistically significant (*p* < 0.05).

## Discussion

Our study found a clear relationship between suffering from various co-occurring chronic physical conditions and poorer distance and near visual performance. Independently, arthritis and stroke were associated with poor visual acuity. With regard to mental health, poor subjective visual acuity was associated with depression. No association was found with anxiety. Both objective and subjective poor VA were associated with worse cognitive performance.

To date, this is the first study that has analyzed the association between suffering from co-occurring physical conditions and the odds of visual impairment, highlighting the additive effect of chronic conditions. After adjusting for covariates, our results show an increasing odds of poor distance and near visual acuity according to the number of chronic physical conditions. Since multimorbidity affects a large proportion of the adult and elderly population, this may be a common risk factor for visual impairment. This is especially important as visual impairment has been related to poorer results in quality of life and disability [3, 6, 51]. The underlying mechanism in this relationship is unknown. By analogy with frailty, in which accumulation of deficits increases vulnerability to adverse outcomes, the co-occurrence of various chronic conditions could be related to a higher risk of visual impairment [52]. People with several chronic conditions, such as cardiovascular conditions, diabetes or arthritis, may have cumulative risk due to vascular, neurodegenerative, biochemical or inflammatory pathways. Some of these relationships have been studied independently in some conditions. For example, diabetes is related to cataracts and diabetic retinopathy, and arthritis has been associated with a higher risk of cataracts [20, 53, 54]. However, it is known that not only diabetes but also hypertension and hypercholesterolemia are risk factors for diabetic retinopathy so, in this case, cumulative effects are possible [55, 56].

Stroke was associated with a high odds of distance and near visual impairment (OR: 1.59 [CI 1.05-2.42]; OR: 3.01 [CI 1.86-4.87]). It is known that stroke is related to a range of visual sequelae, such as low vision, hemianopia, quadrantanopia and motility disorders, but little information is available at an epidemiological level [57]. Low vision may be due to vascular pathology or other ocular abnormalities [58]. Rowe et al. found that up to 92% of stroke survivors have some form of visual impairment [57]. Our results support this relationship and highlight the need for visual assessment after stroke. We also found a higher odds of distance visual impairment in respondents with arthritis. The relationship between arthritis and vision loss is poorly understood and may also be multifactorial. Firstly, extra-articular arthritis symptoms may include uveitis, ulcerative keratitis, scleritis, severe Sjögren syndrome and other conditions directly associated with vision loss [59, 60]. Moreover, medication for arthritis such as corticoids or chloroquine/hydroxicloroquine have been associated with an increased prevalence of glaucoma, cataracts or retinopathy [61–64]. There is, however, no clear evidence on the relationship between osteoarthrosis and visual impairment. At an epidemiological level, there are few data on this issue but it has recently been suggested that there is a higher risk of eye diseases in patients with joint diseases [65]. Finally, individual odds of distance visual impairment was increased in diabetic patients, as expected. However, this association was slight and was not found in the case of near visual acuity, distance subjective visual acuity and near subjective visual acuity. We hypothesize that adjusting by the time from the onset of diabetes could impact the results with diabetes but this information was not available in our study.

With regard to mental health, our results show a clear association between visual impairment and depression. Although visual impairment and depression have been associated in working age adults [66], there is controversy on this topic in older adults [2, 22, 24, 25, 67, 68]. Variability in the studies arises with regard to the type of visual assessment used, including objective visual acuity, medical-record review, presence of age-related eye-disease or vision-loss severity with functional screening. Besides objective measures, the need for measures that accurately assess the degree of visual impairment experienced by the person has been highlighted [69, 70]. Self-experienced visual loss may be important as this could lead to disability, functional decline, and communicative and social isolation [66, 71–74]. In our case, we tested both objective and subjective visual acuity to analyze the nature of their relationships with depression and other mental conditions. We found a stronger association between depression and subjective visual impairment, which highlights the importance of the self-perceived impairment. In fact, the Health Care Policy and Research Cataract Surgery Guidelines suggest that one important indication for cataract surgery may be the degree of functional disability rather than objective visual assessment alone [75, 76]. The differing results in objective and subjective visual impairment with regard to depression could be influenced by the negative perception that these patients may have about themselves. Anxiety showed no association with visual acuity, corroborating previous studies in adults and the elderly [21, 23, 24, 77–79]. There is, however, some controversy as higher rates of anxiety have been observed in populations with some ocular conditions [2, 80]. Our study adds valuable information regarding anxiety because there are relatively few studies on this subject and in most cases they did not have a clear definition of anxiety. Other less-specific variables were used, such as concern about blindness. Moreover, our study deals with objective and subjective visual impairment, providing a general evaluation of the impact of visual performance on anxiety. The relevance of our results is reinforced by the fact that we have used the CIDI questionnaire, a comprehensive, standardized interview that has demonstrated particular usefulness in epidemiological and cross-cultural studies [81]. By using the CIDI, our results can be compared with those of other studies using the same approach.

Finally, cognitive functioning was found to be associated with distance and near visual impairment, at both objective and subjective levels. The strongest relationship was observed in subjective near visual impairment. Mixed results have previously been found with regard to visual performance and cognitive functioning in the elderly population [29–33, 82].

Simulated visual impairment has shown cognitive slowing in adults, especially in the elderly [83, 84]. Moreover, other studies have found a clear relationship between visual performance and cognitive disorders such as Alzheimer’s and Parkinson’s disease [85–87]. The interaction between vision and cognition is not fully understood. On the one hand, visual loss could lead to deprivation of sensory input, leading to cerebral structural or functional changes. On the other hand, a common physiological pathway could be involved. A loss of retinal ganglion cells and impairment of the ventral and dorsal pathways in patients with Alzheimer’s disease has been described [85, 88]. Besides the etiopathogenic implications of the association between vision and cognition, this interaction should be highlighted because coexisting visual and cognitive impairment has been related to a higher risk of disability [89].

Our study has several limitations. Cross-sectional studies identify associations but do not allow cause and effect relationships to be determined. Moreover, age effects may not be distinguished from cohort effects. Longitudinal studies are needed to confirm these results. Multimorbidity research would benefit from standardized inclusion and definition of diseases [65]. In some cases, the Expanded Diagnosis Clusters adapted of the ACG® system have been used, an exhaustive method that is complex to apply outside the clinical setting and where poor integration of health care levels exists [17]. It is known that a higher number of conditions results in a higher proportion of multimorbidity [19]. For our study, the choice of chronic conditions was made according to the SAGE study, focusing on a limited number of conditions that are highly prevalent in the general population and constitute major causes of disability. This methodology allows the work to be conducted across countries. Self-reported data could affect the results, although this would be minimal as acceptable correlation between self-reported and medical-record diagnosis has been found [90, 91]. With respect to vision itself, future studies should include binocular visual acuity assessment and other visual tests such as contrast sensitivity and glare disability. Another limitation is the fact that some differences could arise when using distinct subjective strategies in the visual assessment, which could affect the results [92]. Moreover, since visual impairment becomes more frequent in advanced ages, participants in their fifties could potentially skew the results of the analyses. Consequently, we performed a sensitivity analysis with participants over 65 years which showed similar results compared with the results of the global sample (50 years and over). Finally, poorer results in cognitive functioning may appear in patients with vision loss when the tests include vision tasks [93, 94]. In our study, only the verbal memory tests included a reading part. However, the interviewer read the words aloud if the respondent had reading difficulties, so this bias may be minimized.

## Conclusion

The results of our study contribute to a deeper understanding of visual impairment and its relationship with chronic physical conditions and mental disorders. There seems to be a clear association between a higher number of co-occurring chronic physical conditions and poorer results in distance and near visual functioning. Elderly patients with multimorbidity would benefit from extra eye-care and this would also improve the efficiency of the health care system. At an independent level, suffering from stroke and arthritis is highly associated with poorer results in visual functioning. The presence of these conditions could be triggers for visual impairment. With regard to mental health, poor subjective but not objective visual functioning is closely related to depression, highlighting the importance of subjective perception in depression. Since poor cognition has also been related to poor visual functioning, it would be advisable to monitor mood and cognitive functioning in people suffering from visual disorders.
